# Physiology

**Published:** 1860-07

**Authors:** S. Weir Mitchell

**Affiliations:** Lecturer on Physiology in the Phila. Med. Association


### PHYSIOLOGY.

BY S. WEIR MITCHELL, M.D.,

LECTURER ON PHYSIOLOGY IN THE PHILA. MED. ASSOCIATION.

I.—*Observations on Digestion, made on a Case of Fistulous Opening into the Small Intestine.*

Prof. Busch, of Bonn, has availed himself of the opportunity afforded by a case in which a fistulous opening into the intestine existed, of studying several points in regard to the physiology of the digestive process.

A woman, aged thirty-one, in the sixth month of her fourth pregnancy, was tossed by a bull, one of whose horns lacerated the abdominal wall. At first there seemed to be no injury to the intestine; but at the end of three days a perforation appeared, resulting in the establishment of an opening through which food escaped. In six weeks, the woman, although she ate much, had become extremely emaciated and weak. She was then taken to Bonn. Between the umbilicus and the pubes, there was an aperture in the abdominal wall more than an inch and one-fifth in length, the bottom of which was formed by the posterior wall of the intestine; the upper and lower ends of the intestine were represented by two orifices at the angles of the wounds. The position of the wound, the size of the intestine, the existence of valvulæ connivantes in large number, the fluidity of the chymous material which exuded from the upper end, and its grass-green aspect, led to the inference that the injury had affected the upper third of the small intestine. Nothing whatever passed from the upper to the lower opening; and several attempts to establish a communication proved fruitless. The patient was allowed to eat as much as she wished; but as this was not sufficient, food, such as soups, eggs, etc., was thrown into the lower opening; frequently also, pieces of cooked eggs and meat were thrust into it by the finger. Her general health improved under this treatment; and after a time the supply which she received by the stomach was sufficient.

*Hunger.*—The patient, at first, had a most voracious appetite; she never felt satisfied. She continued to eat even when the first portions of food which she had taken were escaping through the opening. She then would say that she felt better, but still hungry. Prof. Busch infers that hunger is composed of two separate sensations; one general, the other local—the former resulting from the want of material to repair the waste of tissue.

*Movements of the intestine.*—The bottom of the opening was formed of a long portion of the posterior wall of the intestine, the anterior wall having been destroyed by gangrene; there was, also, a large ventral hernia, the coverings of which were so thin, that the least modification in the size of the intestine was as clearly perceived as if it had been laid bare. During the ordinary peristaltic movements, it was not possible to observe any difference between those which took place in the portion of intestine covered by skin, and in that which was exposed to the air. When, on the other hand, the upper part of the intestine became invaginated in the opening, this portion contracted much more actively, and was sometimes the seat of atonic contraction which stiffened and raised it like a solid body.

The intestinal movements were not continuous; there were often intervals of complete rest during more than a quarter of an hour. During these intervals, neither exposure of the part to the temperature of the room, nor the careful introduction of the finger, would excite peristaltic action; even the taking of food would not excite it at once. There was no regularity in these intervals of action and rest, except during part of the night. Up to 10 or 11 P.M. there was a flow of chymous material or of liquids from the upper end of the intestine, so that, at first, the patient was always wet, in spite of all care. No discharge then took place until 4 or 5 o'clock in the morning. When she made a hearty supper, a part escaped at once, but the rest remained in the digestive canal until the next morning. This pause in the intestinal action did not arise from sleep, for it was observed when the patient lay awake at night, and the matters escaped in the daytime while she slept as well as when she was awake.

The escape of the matters contained in the intestine was not continuous, but jerking; and a propulsive movement of the intestine was even observed in the neighborhood of the opening. It is possible, however, that this was only the result of the adhesion of the intestines to the abdominal wall, which furnished a "point d'appui."

Reversed peristalic movements existed and frequently impeded the experiments. Thus the aliments introduced into the lower opening of the intestine were often rejected after some hours, and fatty matters even after some days.

*Observations on the lower end of the intestine; the intestinal juice.*—As the lower end of the intestine received nothing whatever from the upper end, it was possible to study in it the intestinal secretion in its pure state. The quantity of this in the physiological condition was not great. On introducing a bivalve speculum, the intestinal mucus could be plainly seen between the valves; it was white, or of a very slight rose tint. Sugar, tied up in a net bag, and introduced into the intestine, was not completely dissolved in a quarter of an hour. Under pathological irritation the quantity of juice was increased. This was ascertained on two occasions. It was then thick and consistent. The reaction was always alkaline. The quantity of solid matter contained in it varied, according to numerous observations, from 7·4 to 3·87 per cent., the mean being 5·47 per cent.

*Digestive action of the intestinal juice.*—The digestive property of the intestinal juice has been denied by some, while others have, correctly, admitted it. At the commencement of the treatment, the state of the patient began to be improved only when she was fed through the lower opening of the intestine. Into this were introduced soup, beer, gruel, hard eggs, and meat cut into small pieces. During the six weeks previous to her entrance into the hospital, the woman had only two small, hard, stools. After being fed in the way above described, she had at first an abundant evacuation every twenty-four hours; but later it became necessary to use enemata. The evacuations resembled ordinary fæcal matter in form and consistence; but the color was grayish white, and they emitted a repulsive putrid odor. No portions of egg or of meat were found in them.

In order to study the digestive action of the intestinal juice on different foods, a weighed portion of food was inclosed in a net bag, and introduced into the intestine through a speculum. To ascertain the quantity dissolved, the loss of solid matter was sought. For this purpose a known quantity of the same food was dried, and the amount of solid matter estimated, from which, on drying at the same temperature the portion removed in the bag, it was easy to calculate the loss which the latter had sustained.

*Protein matters.*—Coagulated albumen and cooked meat always lost substance, and the morsels when drawn out presented traces of decomposition. The angles of the cubes of albumen were rounded, and the surface had a cheesy aspect, and peeled off in grumous masses when washed with water; the meat was flabby, soft, and pale. The masses had a penetrating, putrefactive odor, and the presence of hydrochloric acid evolved ammoniacal vapor. This was not the result of ordinary putrefaction from moist heat, for none of the experiments lasted above seven hours, and decomposition commenced at an early period. The cause can only be sought for in a peculiar ferment furnished by the intestinal juice. Of albumen the percentage quantity digested varied from 35·35 in six hours and a half to 6·5 in five hours and a quarter; that of meat varied from 29·9 in seven hours to 5·5 in five hours.

*Starch and sugar.*—Starch, dried at 212°, was introduced. The percentage loss varied between 63·53 in five hours and a half, and 38·5 in six hours. After injecting a solution of starch into the lower end of the intestine, the stools were found to contain neither starch nor sugar, but only traces of dextrine. The starch underwent conversion into grape-sugar, which was always found in the bag. Cane-sugar was not changed into grape-sugar; a large portion was found in the fæces in the state of cane-sugar, and the urine contained no trace of it.

*Fats.*—These were submitted to experiment on two occasions. On the first, more than three ounces (avoirdupois) of melted butter were introduced in small portions into the intestine. After ten days the patient spontaneously had a white, very fetid stool, of the consistence of *bouillie.* On being examined, after cooling, it was found to be covered by a layer of solid fat; the subjacent portion, under the microscope, appeared chiefly composed of large fat-drops and fat crystals mixed with epithelial cells. The avacuation had an acid reaction, and the vessel in which it had been treated by ether evolved an odor of butyric acid. A little more than one-sixth of the quantity of fatty matter administered was found. A large portion, however, of the butter had been expelled from the intestine by reversed peristaltic movements, so that probably little or none of it was absorbed. The second experiment, which was made at the end of a fortnight with cod-liver oil, gave precisely the same result.

*Observations on the upper end of the intestine.*—Alimentary matters, contrary to the general supposition, did not remain long in the stomach. In the morning, when the intestine was empty, the peristaltic movements expelled frothy mucus. On giving food which could be readily recognized, the first portions appeared at the opening in about a quarter or half an hour, and after a copious meal, three or four hours were sufficient for the expulsion of the whole.

The reaction of the mixture of digestive fluid, expelled in the fasting state, was almost always neutral, rarely faintly acid or alkaline. The intestinal juice, when carefully washed from the fistulous opening, was constantly found to be alkaline. After a meal, the chymous mass gave very variable reactions. At first, protein compounds seemed to produce an alkaline or neutral liquid, while fat, starch, and sugar gave an acid one; but repeated observations showed so great variations in these respects, that no fixed results could be arrived at.

The solid aliments contained in the chyme did not appear much changed on simple inspection; but on touching the surface of coagulated albumen and pieces of meat, they were found to be more friable than in the fresh state. The muscular fibres of meat were divided both longitudinally and transversely, as is already known to be the case; and this was observed more when the meat had been very finely cut up. These solid alimentary matters floated in a large quantity of biliary fluid; but after the eating of large quantities of cabbage, turnips, and potatoes, a small layer of liquid separated only after the mixture had been allowed to rest a considerable time. Whenever the diet during the day was confined to a single article of food, the chymous mass was more consistent toward evening than in the morning; while after a variety of food, the quantity of liquid was the same at different hours.

After the injection of feculent food, the chyme contained a large amount of starch and sugar; while, after cooked protein compounds, a slight turbidity was very rarely produced on boiling the liquid.

The digestive fluids were rejected in such a mixed state that no definite result could be derived from their examination. The absence of indications of the presence of sulpho-cyanide of potassium showed that saliva was not present. Besides bile and pancreatic juice, there must have been a large amount of fluid supplied by the stomach, for the total of solid matters was very small; the average was 2·48 per cent., the extremes 2·56 and 2·34. This is remarkable, since in man the gastric juice itself gives 3 per cent. of solid residue.

*Cane-sugar.*—After having ascertained that the fluids passed from the opening in the morning, when the patient had been confined to an exclusively animal diet on the preceding day, gave no reaction with the potash and copper test, she was made to take, fasting, solutions of cane-sugar. In the fluid which esaped only a small part of the sugar was found—as grape-sugar, never as cane-sugar. This observation confirms the opinion of M. Bouchardat.

*Raw albumen.*—In the morning, the albumen of four eggs, beaten up with a little water, was given to the patient. After four hours there was collected a moderately large quantity of an alkaline liquid, thready, mixed with bile, and containing no coagulated albumen. If the gastric juice had solidified that substance, portions of it would have been found. The quantity of albumen secreted was found to be 36 per cent. of the amount taken in.

*Gum-arabic* underwent no change, and escaped almost entire from the opening.

*Gelatine.*—Of this substance, nearly two-thirds were absorbed; the remaining third, which escaped, had the ordinary chemical characters, except that it did not gelatinize, and that its warmed solution was rendered turbid by acetic acid.

*Milk.*—After milk had been taken, an acid liquid escaped, in which the casein had been coagulated by the acid of the stomach, not in large masses, but in fine particles. The filtered liquid contained a portion of uncoagulated casein.

*Fats.*—A large dose of cod-liver oil was given to the patient in the morning; and on each occasion the quantity of liquid that escaped was relatively very large. Its reaction varied; most frequently it was acid, rarely alkaline. In the latter case, the fat was so finely divided that it could not be recognized by the naked eye; but, under the microscope, oil globules were found in the molicular state. When the reaction was acid, the greatest part of the fat formed a similar emulsion; but on the top there floated a smaller quantity of oil, in large drops. When the alkaline emulsion had been kept during twenty-four hours, it became acid, and a part of the oil floated on it in large drops.

*Digestibility of certain articles of food.*—Prof. Busch endeavored to obtain information on this subject by examining the quantity of ingested aliment which arrived at the opening in the upper part of the intestine. In one experiment, in which two ounces of sugar were taken, only one-thirtieth part reappeared; in another, in which three ounces were taken, one-fifteenth was found. Of albumen, the proportion of the quantity absorbed to that found discharged in the chyme was as seven to four; of gelatine, almost as two to one.

Prof. Busch has drawn up a table which shows the proportion between the weight of material ingested and that of the chyme thrown out:—

| | Food taken. | Chyme. |
|---|---|---|
| Fat | 1. | 6. |
| Gelatine | 1. | 3·675. |
| Boiled eggs | 1. | 2·73. |
| Meat | 1. | 1·73. |
| Milk | 1. | 1·25. |
| Turnips | 1. | 1·2. |
| Cabbage | 1. | 0·91. |
| Potato soup | 1. | 0·7. |

With regard to the two latter articles, the quantity of juices secreted is too small to make up for the loss of absorption into the stomach and the commencement of the small intestine; while fat and gelatine give rise to a considerable secretion of fluid.

In examining the relation between the quantity of solid matter taken in and that found in the chymous mass, the loss representing the quantity absorbed, the following results were arrived at:—

| | Solid matter taken in. | Solid matter rejected. |
|---|---|---|
| Gelatine | 1. | 0·94. |
| Boiled eggs | 1. | 0·76. |
| Milk | 1. | 0·62. |
| Cabbage | 1. | 0·58. |
| Potato soup | 1. | 0 53. |
| Turnips | 1. | 0·49. |
| Meat | 1. | 0·35. |

Although these numbers cannot be supposed to possess an exact mathematical value, they nevertheless afford important instruction.

Two experiments were made during periods of twenty-four hours: once with a varied animal diet, and at the other time with a mixed vegetable and animal diet. As far as the results of these experiments showed, the amount of matter absorbed from the mixed diet was about ten times as great as that absorbed from the purely animal diet. While the patient was taking the animal diet, the matters which escaped from the opening consisted at first (as always) of a liquid in which floated fragments of meat and of eggs; gradually, however, the quantity of fluid diminished, the mass became more consistent, and at last, especially on the following morning, there escaped a mass having the appearance and smell of pure fresh meat, not colored by bile. Under a mixed diet, the chyme preserved its liquid consistence throughout, except in the middle of the day, when it became more consistent after the patient had taken some legumes at dinner.

*Digestive properties of the fluids discharged from the small intestine.*—In the course of his researches, Prof. Busch observed that the liquid which escaped from the opening, especially after the ingestion of protein aliments, passed very slowly through the filter, and that the fragments of meat and egg continued to diminish in size while lying on the filter, so that at the end of the operation only small portions of solid matter were left. This proved that the digestive influence of the mixture of gastric, pancreatic, and intestinal juices and bile was exerted on the protein compounds from the commencement of the small intestine; and this occurred, whatever was the reaction of the fluid. But the aliment had been in contact with pure gastric juice in the stomach; it was, therefore, interesting to ascertain whether the mixture of digestive juices possessed equal power on food plunged directly into it. Experiment on this point showed that coagulated albumen and roast veal, placed for a period varying from six to eight hours, at a low temperature, in contact with the alkaline fluid which escaped from the fistula after the ingestion of fluid albumen or of meat, lost a small quantity of solid matter and were slightly disintegrated. But this action was incomparably less than that which was exerted by the mixed juices on matter which had been submitted to the action of pure gastric juice. It follows from this, that alimentary matters leave the stomach while imperfectly digested, and that their digestion is performed in great part in the intestines.—(*Archiv. für Pathologische Anatomie und Physiologie*, band. xiv.; and *L' Union Medicale*, 15 et 20 Mars, 1860.)

II.—*Experimental Epicrisis of some late Researches on Liver Sugar.* By J. L. W. THUDICHUM, M.D.

The discussion which has lately taken place at the Royal Society, of a paper by Dr. Harley, in which the views advanced some time ago by Dr. Pavy, and noticed at length in this journal, relative to the glycogenic function of the liver, are controverted, recalls to my memory some experiments which I made in the early months of 1859, with a view of testing the correctness of Dr. Pavy's Chemical Proceedings and Physiological Conclusions. The result of these experiments is adverse to the opinion of Dr. Pavy, and in accordance with that of Dr. Harley, and consequently confirmatory of the doctrine of the glycogenic function of the liver, as generally admitted before the publication of Dr. Pavy's paper. My experiments have, moreover, afforded me an opportunity of ascertaining the sources of fallacy in at least two of Dr. Pavy's modes of proceeding, and have taught me the conditions under which opposite results may be obtained by the same experiment with the same proceeding—a fact which (as I am informed) was unexplained by the late discussion.

A dog of fifteen pounds weight, which had for several days been fed upon tripe exclusively, was narcotized with puff-ball, and deprived of a small piece of his liver. This, without loss of time, was pounded with an equal amount of caustic potassa, without the addition of water; and the pale-reddish magma was filtered through asbestos. The first filtrate, brought into an alkaline solution of copper, gave an abundant red precipitate of suboxide of copper, evidencing the presence of sugar.

Every drop of the filtrate which passed during the next hour yielded the reaction. After that the filtrates ceased to show sugar. The mixture on the funnel had been mixed with water and stirred, and had assumed a dark-red color, having been of a pale flesh-color before.

A second piece of the same liver was exposed to the air for fifteen minutes, and then treated like the first. It exhibited the presence of sugar for one hour, after which the reaction ceased.

The rest of the liver was placed in water boiling in a copper pan. The extract afterwards yielded sugar and glycogenic substance. No leucine was present in this extract.

In the second experiment, a dog of twenty-one pounds weight was narcotized by a blow on the head. One entire lobe of the liver was quickly pounded with potassa; and after incorporation of the two substances, some water was added to the thick magma. It was observed during the pounding and trituration, as well as during the admixture of the water, that the temperature of the mixture rose so considerably, that the hand, which was placed to the outside of the thick wedgewood mortar, felt it to be quite warm. The filtration of this mixture was difficult, owing to the asbestos being too fine. The first clear filtrate was only obtained after half an hour had elapsed, and contained no sugar.

To one ounce of the mixture, ten grains of pure grape-sugar were now added. The filtrate, after twenty-four hours, did not at first give any reaction with copper solution; indeed none at all could be perceived in a test-tube. Only after prolonged standing of the test, in a china dish, the merest trace of red deposit could be distinguished in the bottom of the dish after the fluid had been cautiously decanted. The ten grains of sugar had so effectually been destroyed in the mixture that certainly not one-thousandth part of them was left over.

The rest of the liver contained little glycogenic substance, but no leucine.

The third experiment was made upon a dog of fifteen pounds in weight. The abdomen was opened while the animal was under the influence of puff-ball. The mixture of liver and potash became again very warm, was mixed with water and quickly filtered through cotton by the help of air pressure upon the principle of Beart's coffee machine. The filtrate showed no reaction for sugar, but assumed a violet color.

The first experiment showed me that when a liver containing sugar is treated with caustic potassa, under circumstances preventing any rise of the temperature of the mixture, the sugar, under the influence of caustic potassa and the oxygen of the air and water, is rapidly destroyed.

The second experiment showed that the destruction of sugar, under circumstances favoring a rise of the temperature, is more rapid still, the presence of sugar being, of course, assumed or allowed. It also showed the rapid destruction of sugar, under the influence of potassa and air, at the ordinary temperature.

The third experiment exhibited the influence of a raised temperature alone in destroying (hypothetical) sugar mixed with caustic potassa. So short a time was passed in the experiment, that even allowing some influence to the oxygen dissolved in the water, the influence of the oxygen of the air, though not excluded altogether, must have been limited.

No doubt, in the second and third experiment, the proof of sugar having been originally present in the liver is wanting. But the first experiment made me so well acquainted with all the conditions which are essential for finding or missing any sugar in the liver, by employing potassa as a reagent, that I was quite satisfied of the correctness of the conclusion, which caused me at once to give up all further experiments on this subject, as lost labor; namely, *that no experiment can be admitted as proving the absence of sugar from the liver, in which the contact of that organ with potash, air, and water, at the ordinary or any higher temperature, has not been avoided.*

The behavior of grape-sugar with almost any alkali will for itself substantiate that proposition. A solution of caustic, baryta, and grape-sugar, on standing, is gradually transformed entirely into one of glucate of baryta. A mixture of caustic potassa and grape-sugar, when only heated to 60° or 70° Centigrade, (140° or 158° Fahrenheit,) becomes brown and smells of caramel; and the sugar is entirely transformed into glucic and melassic acids. Grape-sugar fused in the water bath reacts so violently with solution of caustic potassa, that frequently a portion of the mixture is projected out of the vessel employed for the test. Caustic alkali then, of itself, without the interference of oxygen, is most hostile to grape-sugar, and appropriates and transforms it with increasing violence the higher the temperature under which they meet.

A mixture of grape-sugar and caustic potassa is very liable to be attacked by the oxygen of the air, directly and indirectly. Such a mixture, when exposed over night, loses from 30 to 40 per cent. of its sugar, as was proved by Traube, in some experiments made for another purpose. But when to the mixture is added a body having more direct affinity to the oxygen of the air than sugar, but less when that oxygen is once in a bound condition, and therefore easily parting with it, then the oxidizing action of the air becomes most fatal to sugar. Such a body is indigo, which, at the same time, admits of making the experiment visible. It rapidly yields oxygen when dissolved in the alkaline mixture, and is reduced, becoming colorless; but the air restores oxygen and color to the surface, which again passes it to the sugar below. Not until the whole of the sugar is oxidized, does the fluid resume its blue color. The indigo here acts as a mediator between the oxygen of the air and the sugar, by transferring it from one to the other. A precisely similar agent of transfer is the red coloring matter of the blood contained in the liver. When mixed with the alkali, it becomes pale, is reduced; something (sugar when present at all events) is at the same time oxidized. The addition of water containing oxygen, the contact of the air itself, restores the dark-red color, immediately to be lost again in the lower strata of the fluid. Ultimately the fluid remains dark; all oxidizable matter is destroyed. The sugar of the liver is not only destroyed by the direct agency of air and caustic potassa, but also by the oxygen, which the red coloring matter of the blood has a remarkable power of binding and transferring. When I applied these considerations to the experiment of Dr. Pavy, in which he injects a solution of caustic potash (in water not stated to have before been freed of oxygen) into the liver of animals, (showing the temperature of 37° Centigrade, or 99° Fahrenheit, and being saturated with oxygen, or filled with blood, containing it in a loosely combined state,) I was forced to the conclusion that any sugar which might be present would assuredly be oxidized and destroyed at once.

The hurry with which Dr. Pavy found himself obliged to operate, to avoid finding any sugar, has its ready explanation in the rapid decrease of the temperature which the liver does experience when the abdomen is opened. The higher the temperature at which sugar, potash, and oxygen meet, the more rapid and complete is the destruction of sugar; if the liver is allowed to get a little cool before injecting the potash, sugar is found, because it has not all been destroyed.

The facility with which sugar in alkaline solutions attracts oxygen from the oxydes of metals points the same way. The reaction with copper has become *the* test for grape-sugar. Acetate of copper, peroxyde of lead, nitrate of suboxyde of mercury, corrosive sublimate, nitrate of silver, chloride of gold, trisnitrate of bismuth, surrender a portion or the whole of their oxygen to grape-sugar in alkaline, neutral, or even acid solutions. The avidity of grape-sugar for oxygen, under a great variety of circumstance, is really astounding.

The potash test having been the basis of Dr. Pavy's conclusions, and being now proved to be fallacious, the conclusions themselves, in so far as they are derived from the analysis with potash, are not any longer valid. The reverse of Dr. Pavy's results has moreover been obtained by means of my own; and now, by the much more extensive experiments of Dr. Harley, made with Dr. Pavy's own method. The experiments in which freezing and citric acid are employed respectively, I have not repeated. But this I have experienced in some preliminary tests, that alkaline citrates have so strong an affinity for the oxygen of the air, that red suboxide of copper, lying at the bottom of a test-tube half filled with alkaline citrate, after exposure to the air for some days, becomes oxidized again and dissolves with a blue color in the previously colorless fluid.

I must confess that I am unable to comprehend the meaning of the freezing experiment; but leaving aside speculation, there is in Dr. Pavy's proceedings following the freezing, much reason for distrusting that experiment also. He met with great difficulty in filtering his alkaline solutions of liver, and therefore neutralized them with sulphuric acid before filtration, filtered them by squeezing through flannel, and then again made them alkaline. The mixture thereby not only became enormously bulky, but was for a length of time exposed to the air under a variety of circumstances; Dr. Pavy apparently taking it for granted that when liver and potassa were once incorporated, the rest of the operation could be completed at leisure, as sugar would not any longer be formed. But in absence of the guarantee that none would be destroyed, and in presence of evidence that much is actually destroyed under those conditions, his very security makes it doubtful whether he employed even that moderate speed which his method permitted.

For the repetition of Dr. Pavy's, or other similar experiments, I can recommend a small Beart's coffee machine, as the speediest and best contrivance for effecting quick filtration.

As the result of many experiments, Dr. Pavy found that there was no sugar present in the liver; but on the other hand he gives, as the result of sixty observations, that sugar is only found to the extent of the merest trace in the blood of the right side of the heart, under a natural or ordinary condition, during life. Even this result, although of a positive nature, and speaking against his conclusions relative to the liver, is not without objections, based upon the mode of analysis employed. He defibrinated the blood, probably by stirring, treated it with spirit, and pressed through flannel. The residue of the evaporation was tested with Barreswil's solution.

Blood, to be analyzed for sugar, in order to find a maximum, must be analyzed much as for urea; it must be allowed to flow directly into absolute alcohol, and afterwards the additional precaution must be taken of removing its alkalinity when the filtrate concentrates on the water-bath, otherwise much sugar is necessarily destroyed.

I had formed the above positive opinions and conclusions on Dr. Pavy's experiments many months ago, as many friends can testify, who were either present at one or the other of the experiments, or were in conversation informed of my conclusions. For this reason my results and reflections, which were obtained quite independently, may be considered a strong confirmation of some of the propositions of Dr. Harley, as contained in the published abstract of his paper. —(*British Medical Journal*, London, Saturday, March 17, 1860.)

III.—*The Coccygeal Gland of Man.* By PROFESSOR LUSCHKA, of Tubingen.

The coccygeal gland is situated, as its name indicates, on the floor of the smaller pelvic basin. It was discovered by Professor Luschka while studying the anatomy of this region. It has no great interest in connection with the topographical anatomy of these parts; but its existence would seem to cast light on some questions in embryogeny and pathological anatomy. From this point of view, therefore, the discovery of Professor Luschka merits attention.

This gland, whose existence is constant, is a single organ, about the size of a pea, oval, yellowish red, and of a rough surface. It is situated immediately in front of the summit of the coccyx, in a kind of median grove, between the two tendinous insertions of the levator ani muscle, at about the fourth division of the coccyx; in front, it is covered by the fibres of the retractor of the anus* and its aponeurosis; behind, it is in relation with the coccygeal insertion of the anal sphincter. The gland is best found from its posterior side, by dissecting away and removing successively the skin and the sphincter of the anus. As it is ordinarily situated in a rather abundant atmosphere of fat, it is better at first to make this dissection on thin subjects.

Not uncommonly the coccygeal gland is found to consist of five or six separate granules, the size of millet grains, suspended in cluster on the smaller branches of the middle sacral artery, and reunited to one another by areolar tissue. It resembles a good deal the granular bodies so frequently found in the neighborhood of the kidneys in certain cartilaginous fish, (choudropterygians,) and which, according to Stannius, appear to be the analogues of the supra-renal capsules. The same granular character is discoverable even when the gland seems at first sight to be formed of one homogeneous mass.

The parenchyma of the coccygeal gland is always of considerable consistence. For microscopic examination it should be torn with needles or thin sections of

* A portion of the levator ani muscle.

it made with the aid of scissors; most of the structural details become more distinct upon the addition of acetic acid. This procedure enables us to distinguish in the gland structure, on the one hand, a dense stroma of connective tissues rich in nuclei, and on the other, vesicular interspaces and culs-de-sac inclosed in the areola of the stroma.

The vescicles, whose diameter varies from the four-hundredth to the twelve-hundredth of a millimetre, are disseminated in less or greater number among the other elements. In their exterior appearance they resemble the ductless follicles of the digestive tube; being distinguished from these, however, by the absence of vessels and of an areolar stroma in their interior.

The culs-de-sac are very irregular in form and arrangement. They are more or less twisted and contorted in various ways, and here and there are very much narrowed. Ordinarily simple, they are at other times furnished with appendages whose form varies much, or again, they may be found to ramify in such away as very exactly to simulate the arrangement of the conglomerate glands. They are never found, however, to reunite in a common excretory conduit.

These culs-de-sac, as well as the vesicles, are formed by a basement membrane, which is hyaline, structureless, and more or less confounded with the fibrous stroma and its cellular elements. There are many variations in the form and arrangement of these latter elements. Most generally, the internal face of the basement membrane is lined with a layer of nucleated cells, analogous to glandular epithelium; at other times these cells are of irregular shape, and are more like certain epithelium elements of the choroid plexus. The rest of the cavity is filled up by an amorphous mass, in which are disseminated a great number of nuclei and nucleated cells, the epithelium covering having disappeared. Finally, in the very young, some of the vesicles occasionally present cylindrical epithelial cells, furnished in some cases with vibratile cilia.

The coccygeal gland is supplied by many small vessels, which come off from the middle sacral artery, and distribute themselves in a loose capillary net-work around the vesicles and the culs-de-sac. An enormous number of nerves also enter the gland; they arise from the coccgyeal ganglion, from the ganglion impar, or, this being absent, from the inferior looped extremity formed by the union of the two great sympathetic nerves. These nerve fibres form a close plexus in the parenchyma of the gland; in this situation can often be seen primitive nerve cylinders ending in the slight enlargement. These extremities appear to carry a ganglion cell, such as are found, according to Kölliker, almost constantly among the branches of the last sacral nerve.

In structure the coccygeal gland resembles the glands known as the ductless glands. It is very remarkable that it should be found in a situation which corresponds to the lower extremity of the dorsal chord, just as at the other of this great nervous trunk is to be found the greater lobe of the pituitary gland, these two bodies being thus placed in the same relation with the two extremities of the great sympathetic nerve.—(*Gazette Hebdomadaire*, April 20, 1860; from the *Archiv für Pathologische Anatomie*, viii., new series, p. 106.)

